# The power of simplicity: a fast-and-frugal heuristics approach to performance science

**DOI:** 10.3389/fpsyg.2015.01672

**Published:** 2015-10-29

**Authors:** Markus Raab, Gerd Gigerenzer

**Affiliations:** ^1^Department of Performance Psychology, Institute of Psychology, German Sport UniversityCologne, Germany; ^2^Sport and Exercise Science Research Centre, School of Applied Sciences, London South Bank UniversityLondon, UK; ^3^Center for Adaptive Behavior and Cognition, Max Planck Institute for Human DevelopmentBerlin, Germany

**Keywords:** sports, medicine, simple heuristics, take-the-first heuristic, fast-and-frugal trees

## Abstract

Performance science is a fairly new multidisciplinary field that integrates performance domains such as sports, medicine, business, and the arts. To give its many branches a structure and its research a direction, it requires a theoretical framework. We demonstrate the applications of this framework with examples from sport and medicine. Because performance science deals mainly with situations of uncertainty rather than known risks, the needed framework can be provided by the fast-and-frugal heuristics approach. According to this approach, experts learn to rely on heuristics in an adaptive way in order to make accurate decisions. We investigate the adaptive use of heuristics in three ways: the descriptive study of the heuristics in the cognitive “adaptive toolbox;” the prescriptive study of their “ecological rationality,” that is, the characterization of the situations in which a given heuristic works; and the engineering study of “intuitive design,” that is, the design of transparent aids for making better decisions.

## Introduction

An important aspect of performance science as a new discipline is to describe, explain, predict, and change human behavior. Consider the German player Mario Götze’s 2014 World Cup goal in the final match between Germany and Argentina. Describing his performance in terms of his technical and tactical skills and explaining his behavior by looking back at his previous successes and his time in talent development programs are typical research strategies in movement and sport science (e.g., de Oliveira et al., 2014). But predicting that Mario Götze would score in the final game is the kind of thing on which a betting market earns billions per year; most people fail even to predict the final score of a competition without the luck of a good guess (e.g., Lessmann et al., 2010). Identifying and training young talent in a way that increases the likelihood of their success in adulthood is another challenge faced in this domain (e.g., Suss and Ward, 2014).

Consider a second example from medicine, when a doctor needs to decide whether a patient with severe chest pain should be allocated to the coronary care unit (CCU) or to a regular nursing bed. Finding the answer is often accomplished with a fixed set of procedures such as a sequence of diagnostics but also by the doctor’s experience and intuition (e.g., Wegwarth et al., 2009). Once again, it is much more difficult to predict (in this case, whether a patient with chest pain will have a heart attack over the next few days) than to explain in hindsight why it occurred.

The distinction between hindsight and foresight has its parallel in the distinction between “risk” and “uncertainty.” In situations of risk, all possible alternatives are known, as are all possible consequences and their probabilities (Knight, 1921). Classical decision theory is designed for situations under risk such as monetary gambles and lotteries, where probability theory suffices for making decisions. This level of certainty is rare, however, in sports and medicine. The two fields abound with situations of uncertainty, where not all alternatives, consequences, or probabilities are known, and where probability theory alone is thus of little help. In these situations, useful tools for dealing with uncertainty are heuristics (Gigerenzer and Gaissmaier, 2011; see **Table 1** in this paper for heuristics). Because performance science generally deals with situations of uncertainty as opposed to calculable risk, we argue that the study of fast-and-frugal heuristics can provide a conceptual framework (Gigerenzer et al., 2011)^1^.

**Table 1:** Heuristics applied to medicine and sport.

Heuristic	Definition	Ecologically rational if	Bold predictions	Example
Recognition heuristic (RH) Goldstein and Gigerenzer, 2002	If one of two alternatives is recognized, infer that it has the higher value on the criterion.	Recognition validity >0.5	Contradicting information about recognized object is ignored, less-is-more effect if a >b, forgetting is beneficial	RH predicted the winners of the Wimbledon tennis matches better than the predictions by Wimbledon experts’ seeding and ATP rankings Serwe and Frings, 2006; Scheibehenne and Bröder, 2007
Take-the-best Gigerenzer and Goldstein, 1996	Infer which of two alternatives has the higher value by (a) searching through cues in order of validity, (b) stopping the search as soon as a cue discriminates, (c) choosing the alternative this cue favors.	Cue validities vary highly, moderate to high redundancy, scarce information	Can predict as accurately as or more than multiple regression, neural networks, exemplar models, and classification and regression trees.	Professional burglars’ choice of location to break-in is predicted more accurately by take-the-best than by a weighted-linear strategy. For novices’ choice, the opposite holds Snook et al., 2011
Take-the-first Johnson and Raab, 2003	Choice from self-generated options by (a) searching through options in order of validity (b) stopping search after two to three options (c) choosing the first option generated	Option validity vary highly, option validity is learned through feedback	Can predict limited search better than memory models Raab, 2012	Handball playmakers allocations follow it Raab and Johnson, 2007
Hot-hand heuristic Csapo et al., 2015	If one of two alternatives has a positive streak of success, infer that this option has a higher probability of being successful again	Base rates are unknown or vary, correlation between sequential shots are performance is positive	Can predict choices better than models that ignore the sequential dependence of choices Raab et al., 2012, can perform better than base-rate models Burns, 2004	Basketball coaches and players use it Raab et al., 2012; Csapo et al., 2015
Fast-and-frugal tree Green and Mehr, 1997	Classify an object into two categories by (a) searching through cues according to their order, (b) stopping the search as soon a cue allows to do so, and (c) choosing the object the exit specifies (see **Figure 1**)	See take-the-best heuristic.	Can predict as accurately as or better than logistic regression Wegwarth et al., 2009	A fast-and-frugal tree predicted heart attacks better than the Heart Disease Predictive Instrument (HDPI; a logistic regression) and physicians.

## The Fast-And-Frugal Heuristics Approach

The study of heuristics has three goals. The first is descriptive and looks at the question of which heuristics people use. Answering it requires analysis of the “adaptive toolbox” (collection of heuristics) that individuals have at their disposal, including how the heuristics in the toolbox are learned and applied. The second goal is prescriptive and concerns the question of when one should use which heuristic. Examining this is known as the study of the ecological rationality of heuristics. The final goal is one of engineering, called “intuitive design,” that is, the design of heuristic tools and/or environments that improve decision making (Gigerenzer et al., 2011). Each of these three goals is relevant for performance science: to understand the heuristics experts rely on, to understand in what situations a heuristic is likely to be successful, and to design expert systems that improve decision making.

Let us first explain what exactly a heuristic is. A heuristic is composed of building blocks, typically three: search rules that specify where to look for information, stopping rules that specify when to end search, and decision rules that specify how to make a final decision. For instance, consider the task of predicting the winners of the 127 matches (with 128 contestants) of the Gentlemen’s Singles Championship in Wimbledon, situations of high uncertainty. A “rational” strategy might be to use the ATP rankings or the experts’ seeding and predict that the higher ranked player will win each game. A heuristic approach to predicting the winners might instead rely on the recognition heuristic (RH): if you have heard of one player, but not the other, predict that the recognized player will win. The RH comprises a search rule (retrieve recognition information from memory), stopping rule (stop search immediately thereafter), and decision rule (go with the recognized object). Two studies showed that this simple heuristic actually predicted the winners on average better than the ATP rankings and the Wimbledon experts’ seeding (Serwe and Frings, 2006; Scheibehenne and Bröder, 2007). The RH works (i.e., is ecologically rational) when a positive correlation between the recognized object and a target value such as the competition strength of a player exists, which is often the case in sports (see Goldstein and Gigerenzer, 2002, for formal descriptions of the conditions). It is not ecologically rational in situations where no such correlation exists. In other words, a heuristic is not accurate or inaccurate per se, but its rationality is co-determined by the environment. Experimental studies show that in these situations, people no longer rely on recognition and switch to other available heuristics, such as take-the-best (see **Table 1**).

The RH is a truly simple heuristic, and people rely on it for various choice tasks (“go with what you know”). In performance science, researchers may not only be interested in choice but also in how that choice is translated into action. In soccer, for instance, it is of equal interest how a player arrived at the decision to attempt a goal and whether the shot was successful. Thus, one aim is to train players to be capable of shooting to the goal in different ways and situations. Decisions on what (e.g., shoot to the goal) and how (e.g., curved ball around a defender in the upper left corner of the goal) are considered separately. In terms of heuristics, different building blocks might focus on the decision and its execution (Raab et al., 2005).

In most of these cases, the aim of research is to improve performance through better diagnostics and intervention. This sounds logical but is not always a simple task. For instance, out of 1 million young soccer players under the age of 18 in a talent development program such as the German Soccer Association it is difficult to identify those 700 players who will be recruited by young talent centers throughout Germany and predict which of them will be drafted by the highly selective premier leagues some years later. A similar level of uncertainty is evident in medicine, where technology provides doctors and patients with huge amounts of data but where fast decisions are often needed to stabilize a patient’s health. The simple heuristics approach provides a framework for performance science to systematically analyze how to improve performance.

## Heuristics in Performance Science

Performance science, as a multidisciplinary field focusing on human performance under various conditions, may benefit from applying the simple heuristics approach to any of its domains. Here we focus on examples of heuristics in sports and medicine. These heuristics can be adapted to other fields as well (see the articles in Gigerenzer et al., 2011), and thus provide best practice examples of an approach that can lead to research innovation.

### Heuristics in Sports

For an overview of simple heuristics applied in sports, see Raab (2012). A number of these heuristics, such as the RH described above, were discovered and modeled in cognitive psychology, behavioral biology, and other fields and summarized in special issues (Marewski et al., 2010, 2011a,b). Others, however, such as the hot-hand heuristic, have their origin in sports. This heuristic reflects the hot-hand belief held by many athletes, coaches, and fans that a player who has just scored two or three hits in a row has a higher chance of scoring again than if that player had just had two or three misses in a row. Earlier research claimed that sequences of hits and misses are independent and that the hot-hand belief is thus a fallacy (e.g., Gilovich et al., 1985). Yet recent reviews (Bar-Eli et al., 2006; Avugos et al., 2013) show a more mixed pattern of results, and a study using large data sets suggested that at least some players have successful streaks (Csapo et al., 2015). In response to those calling the hot-hand belief a fallacy, critics have argued that the defense will attack a “hot” player and thus prevent streaks from occurring. Indeed, in volleyball, where the net limits direct counterattack, a “hot hand” was found for half of the players (Raab et al., 2012). Moreover, coaches and playmakers were able to detect players’ local improvements (the hot hand) and use them to make strategic decisions. That is, playmakers were using a “hot-hand heuristic” to decide to whom to allocate the ball (see also Bennis and Pachur, 2006; Csapo et al., 2015).

The hot-hand heuristic is effective in some environments, such as when base rates are not reliable or not known or when base rates and hot streaks are highly correlated, as demonstrated in the Raab et al. (2012) study in volleyball. No streak in sports is endless, and thus athletes or fans use such knowledge when they predict whether a streak will continue or not (Raab and MacMahon, 2015). Further, even false beliefs can have a beneficial effect on performance, as empirical evidence has shown (Burns, 2004; Gula and Raab, 2004; Raab et al., 2012).

A final illustration of a heuristic that players rely on in sports is the take-the-first heuristic, which explains how experienced players choose an option (such as shoot or pass). The heuristic uses a search rule that generates options (from memory or external sources) in the order of their validity. Option validity is based on previous experience such that the option that would allow the highest probability of success is generated first. Search is stopped after this first feasible option is generated, hence the name “take-the-first.” Empirical evidence supports this description of option generation for elite players in different sports (e.g., team handball, Johnson and Raab, 2003; basketball, Hepler and Feltz, 2011) and suggests that the tendency to pick the first option is influenced by situational conditions (Belling et al., 2015), the emotional state of the decision maker (Laborde and Raab, 2011), and personality characteristics (Raab and Laborde, 2011). Furthermore, gaze behavior has been shown to match option generation (Glöckner et al., 2012), and longitudinal studies have shown how the take-the-first heuristic is learned on the road to expertise (Raab and Johnson, 2007). The take-the-first heuristic has been compared to alternative models such as the long-term working memory model applied in North et al. (2011), which proposes a positive relation between the number of generated options and performance, whereas take-the-first predicts a negative relation between these factors. Since its discovery (Johnson and Raab, 2003), the take-the-first heuristic has been transferred to other domains of performance science, including nursing (Whyte et al., 2009), navigation (Conlin, 2009), law (von Helversen and Rieskamp, 2009), medicine (Wegwarth et al., 2009), consumer choice (Nordgren and Dijkstershuis, 2009), and engineering (Simpson, 2008).

### Heuristics in Medicine

Simple heuristics have been applied to medicine for decades, but only recently has the power of simplicity been systematically studied. For instance, in emergency rooms, doctors routinely have to decide whether to send a patient with chest pain to the CCU or a regular nursing bed. Doctors facing this decision in one Michigan Hospital relied on defensive decision making—that is, protected themselves against potential lawsuits—and sent about 90% of the patients to the CCU even though only about 25% of them actually had a coronary problem that warranted being sent there (Green and Mehr, 1997). As a consequence, the units became overcrowded, the quality of care declined, and medical costs increased. One attempted solution was to provide doctors with the Heart Disease Predictive Instrument (HDPI), a chart with some 50 probabilities and a pocket calculator with a logistic regression to determine the probability that the patient should be sent to the CCU. The HDPI made better allocations than physicians’ defensive decision making, but physicians’ disliked using a tool that they did not understand.

**FIGURE 1:**
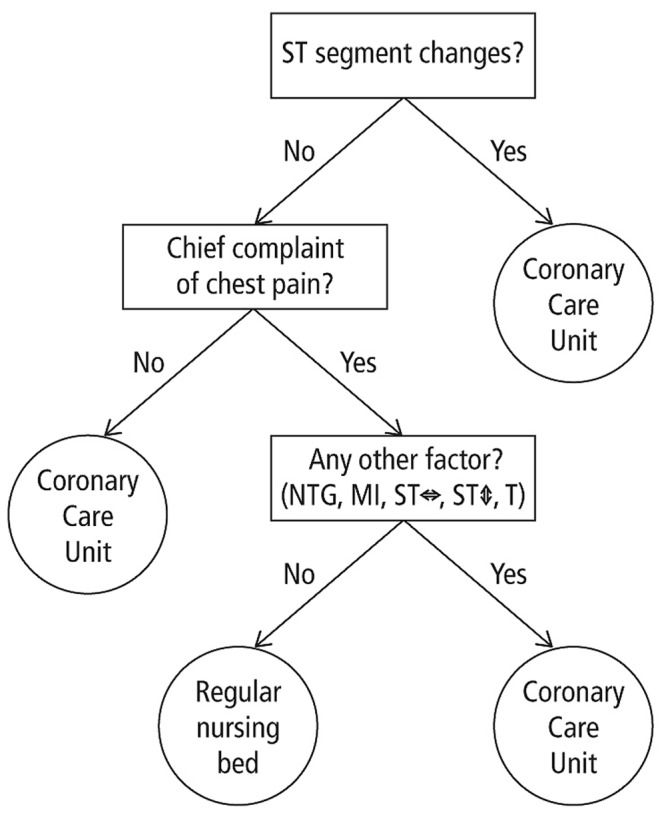
**Fast-and-frugal tree for emergency physicians to detect acute ischemic heart disease.** It only asks up to three yes/no questions, namely whether the patient’s electrocardiogram shows a certain anomaly (“ST segment changes”), whether chest pain is the patient’s primary complaint, and whether there is any other factor (Based on Green and Mehr, 1997).

Inspired by the take-the-best heuristic, two researchers from the University of Michigan developed and implemented a fast-and-frugal decision tree to replace the HDPI (Green and Mehr, 1997). The tree asks three questions only (**Figure 1**): “Is there a certain anomaly (ST segment changes) in the electrocardiogram?” “Is chest pain the chief complaint?” and “Are there are any other factors present such as myocardial infarction or nitroglycerin use for chest pain relief?” Unlike a full decision tree with n^2^ exits (n = number of questions), a fast-and-frugal tree has n +1 exits, with one exit at each question and two at the final question. Thus, it allows decisions to be made faster and with limited information, and is intuitively understandable (intuitive design). But how accurate is it? A study at the hospital reported that it led to fewer misses and a better false-alarm rate than both the HDPI and physicians’ decisions, the latter of which were even slightly below chance (Green and Mehr, 1997). The fast-and-frugal tree is still used by the physicians, who can easily adapt it to new patient populations because they understand its structure (Wegwarth et al., 2009).

## Simple Heuristics: A Framework for Performance Science

Sports and medicine are not the only areas where fast-and-frugal heuristics have been investigated or implemented. The role of heuristics in the making and execution of law is discussed by Gigerenzer and Engel (2006) and Kelman (2011). An entire issue of the *Journal of Business Research* (2014, 67) was devoted to the topic of risk, uncertainty, and heuristics (see Mousavi and Gigerenzer, 2014). Management and interview processes are covered by Gigerenzer (2006, 2014), Fific and Gigerenzer (2014), and Brighton and Gigerenzer (2015). Recently the Bank of England started a program called “simple heuristics for a safer world of finance” (Aikman et al., 2014). More examples can be found in overviews of foundations and applications of heuristics provided by Gigerenzer and Gaissmaier (2011), Gigerenzer et al. (2011), and Todd et al. (2012).

In this short paper, we argued that performance science deals to a large degree with situations of uncertainty, as opposed to situations of calculable risks. Under uncertainty, probability theory is no longer sufficient for making good decisions. As we show, however, heuristics are tailor-made to deal with limited predictability. **Table 1** provides a few examples of heuristics from the adaptive toolbox of individuals. The study of the ecological rationality of heuristics answers the question of when a given heuristic will be successful or not compared to other strategies. Further, we argued that intuitive design refers to the use of insights from the study of the adaptive toolbox and ecological rationality in order to engineer decision tools (or environments) conducive to making better decisions. By design, these tools are intuitive and can thus be easily learned and adapted. We believe that the fast-and-frugal heuristics approach as a framework can aid performance science in developing a structured research program.

## Conflict of Interest Statement

The authors declare that the research was conducted in the absence of any commercial or financial relationships that could be construed as a potential conflict of interest.

## Footnotes

1 This approach is based on the pioneering work by Herbert Simon emphasizing the need for formal models of heuristics and their analysis under conditions of uncertainty as opposed to risk, unlike traditional approaches to heuristics (e.g., Tversky and Kahneman, 1974). The heuristic concept applied here is based on Gigerenzer et al.’s (2011) notion of fast-and-frugal heuristics. For a book-length discussion of these two programs from a legal perspective, see Kelman (2011).
